# Adopting the Joint Line Theory for Bone Resection in Cruciate‐Retaining Total Knee Arthroplasty to Prevent Flexion Gap Tightness

**DOI:** 10.1111/os.13256

**Published:** 2022-04-18

**Authors:** Ken Okazaki

**Affiliations:** ^1^ Department of Orthopaedic Surgery Tokyo Women's Medical University Tokyo Japan

**Keywords:** Joint line, Osteoarthritis, Posterior cruciate ligament, Surgical techniques, Total knee arthroplasty

## Abstract

**Background:**

During a conventional measured resection using the posterior reference method for total knee arthroplasty (TKA) in varus knees, proximal tibia is resected from the lateral joint surface for the same thickness as the implant. Distal femur is resected from the worn medial surface for the same thickness as the implant. Posterior femur is resected using the posterior reference method with an external rotation for appropriate degrees. In this situation, although the joint line of the tibia is leveled to the height of lateral joint surface, the posterior joint line of the femur is leveled to the center of medial and lateral posterior condyle, which is a few millimeters lower than the lateral posterior condyle. This discrepancy between the proximal tibia‐posterior femoral joint line causes a tight flexion gap in cruciate‐retaining TKA. Therefore, downsizing of the femur is necessary to adjust the posterior joint line to the level of the lateral condyle.

**Perspectives:**

To avoid this circumstance, the postoperative joint line should be leveled to the center of the original medial and lateral joint surface. Proximal tibia is resected from the lateral joint surface 1 mm to 2 mm thicker than the implant. Distal femur is resected from the worn medial surface 1 mm to 2 mm thinner than the implant. Posterior femur is resected using the posterior reference method with an external rotation for appropriate degrees. In this situation, all the joint lines are leveled to the center of the medial and lateral joint surface. Otherwise, use of an anatomically shaped implant with a physiologic joint line is another option to avoid joint line discrepancy.

**Conclusions:**

Adopting joint line theory for bone resection can prevent the flexion gap tightness that likely occurs in cruciate‐retaining TKA.

## Graphical abstract

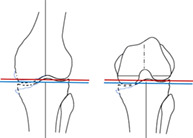

## Introduction

A tight flexion gap is often encountered during cruciate‐retaining (CR) total knee arthroplasty (TKA). Methods to manage tension of the posterior cruciate ligament (PCL) have been frequently described, such as PCL release and the pie‐crusting method^1^, ^2^. Furthermore, some CR‐TKA systems have an option in which the posterior condyle is smaller by approximately 2 mm and is changeable after bone preparation during surgery (e.g. NexGen CR‐Flex; Zimmer Biomet, Warsaw, IN, USA). Reportedly, the insufficient posterior inclination of the tibia is one reason why the flexion gap becomes narrow^3^, ^4^. In fact, a previous study showed that the difference of posterior tibial inclination by 5° influences the flexion gap by approximately 2 mm with CR‐TKA^5^. However, surgical error in the posterior inclination of 5° is considered rare. To prevent a tight flexion gap during CR‐TKA, more attention should be paid to alteration of the joint line at the medial and lateral sides on the proximal tibia, distal femur, and posterior femoral condyle.

## Joint Line Theory

**Fig. 1:**
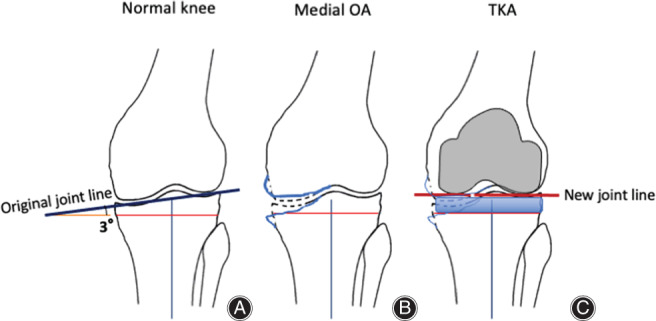
Joint line alteration in coronal plane. (A) Schema of the normal knee. The original joint line (black line) is medially inclined relative to the mechanical axis. The average angle is 3°. (B) Schema of medial OA knee. Cartilage and subchondral bone at the medial side are worn. (C) Schema of total knee arthroplasty. When the proximal tibia and distal femur are resected at the same thickness as the implants from the lateral surface, the joint line after implantation (*redline*) is horizontally leveled to the lateral joint line height.

**Fig. 2:**
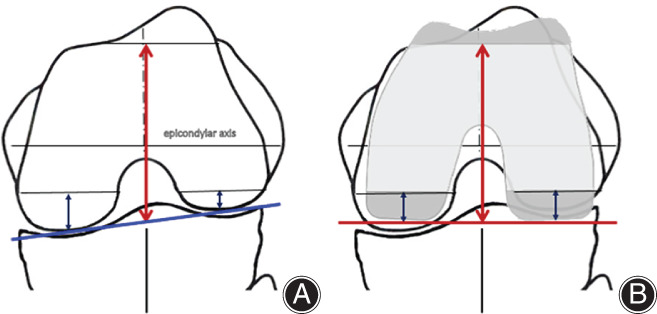
Joint line alteration in axial plane. (A) Schema of the knee in flexion. The original posterior femoral joint line is indicated by the blue line. The anterior–posterior size is usually measured at the center of the medial and lateral condyles (red arrow). When the femoral component is aligned to the epicondylar axis, posterior condyle bone resection (black arrow) is thicker in the medial condyle and thinner in the lateral condyle. (B) Schema of total knee arthroplasty using the posterior reference method. When the external rotation is 3° relative to the original posterior joint line, the new posterior joint line after implantation (red line) is approximately 1.5 mm anterior at the medial side and 1.5 mm posterior at the lateral side, relative to the original joint line. Note the new joint line is lower than the height of the lateral surface of the tibia.

The joint line of the normal knee is medially inclined an average of 3° relative to the mechanical axis. After TKA, the joint line is altered to be perpendicular to the mechanical axis with the conventional mechanically aligned method. Many operations using the standard measured resection method indicate that the proximal tibia should be excised with the same thickness as the tibial implant from the lateral normal articular surface (e.g. 10 mm). In that case, the joint line after implantation is leveled to the height of the original lateral joint surface (Fig. 1). The medial side is elevated by approximately 2–3 mm from the native medial joint line before the onset of osteoarthritis (OA). The distal femur is usually excised with the thickness of the implant from the worn medial joint or lateral healthy cartilage surfaces. As a result, the joint line at the distal femur is also elevated 2 to 3 mm above the original medial joint line. Regarding the posterior femoral condyle, cartilage often remains. Moreover, most femoral sizing guides measure the anteroposterior width at the center of the medial and lateral posterior condyles (Fig. 2A). For example, after the posterior reference method with 3° of external rotation, the medial posterior condyle becomes approximately 1.5 mm smaller, and the lateral posterior condyle becomes approximately 1.5 mm larger (Fig. 2B). On the other hand, the tibial joint line is leveled to the lateral joint surface, which is higher than the original medial joint surface by approximately 3 mm. A simple calculation shows how the flexion gap becomes as narrow as 1.5 mm.

### How to Avoid a Tight Flexion Gap in CR‐TKA

There are two options for adjustment of the postoperative joint line.

**Fig. 3:**
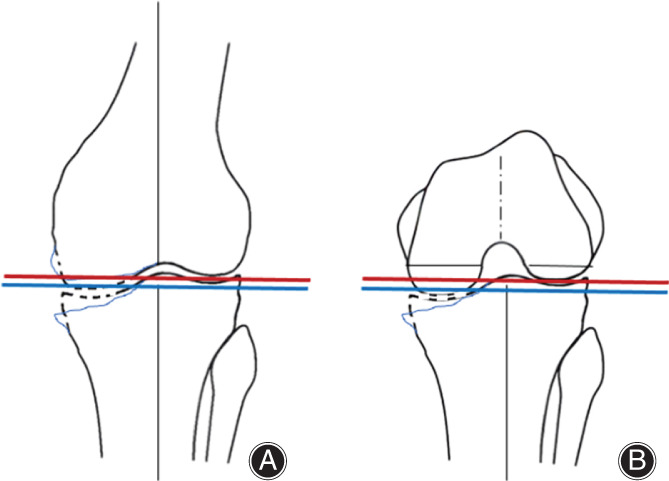
The option of joint line setting after total knee arthroplasty in extension (A) and flexion (B). The blue line is leveled to the height of the center of the medial and lateral joint surfaces (method 1). The red line is leveled to the height of the lateral joint surface (method 2).

In the first option, adjust the joint line to the height between the medial and lateral joint surfaces, not to the level of the lateral joint surface (Fig. 3, blue line). In detail, the lateral joint line is set approximately 1.5 mm lower and the medial joint line approximately 1.5 mm higher than the native lateral joint surface and medial joint line, respectively. For femoral sizing, when the posterior reference method is used with the sizing guide that is measured in the center, the joint line will be aligned in the center of the medial and lateral posterior condyles. The medial posterior condyle is approximately 1.5 mm smaller, and the lateral posterior condyle approximately 1.5 mm larger.

Because the medial side is worn in the case of medial OA, the original medial joint line is no longer detectable, especially with a severe deformity. Therefore, the lateral intact cartilage is suitable for the reference. The proximal tibia is cut from the lateral healthy articular cartilage surface, 1 mm to 2 mm thicker than the tibial implant to be used (e.g. 11 mm for a 10‐mm implant). The distal femur is cut from the lateral articular cartilage surface, 1 mm to 2 mm thinner than the thickness of the implant (e.g. 8 mm for a 9‐mm implant). Therefore, the tibia joint line and the distal femoral joint line are set approximately 1 mm to 2 mm lower than the lateral joint surface. The femoral posterior condyle resection is determined by the posterior reference method, using a sizing guide measured at the femoral center. By doing so, the entire joint line is set to the center level between the medial and lateral joint levels. Therefore, the risk of a tight flexion gap can be avoided.

**Fig. 4:**
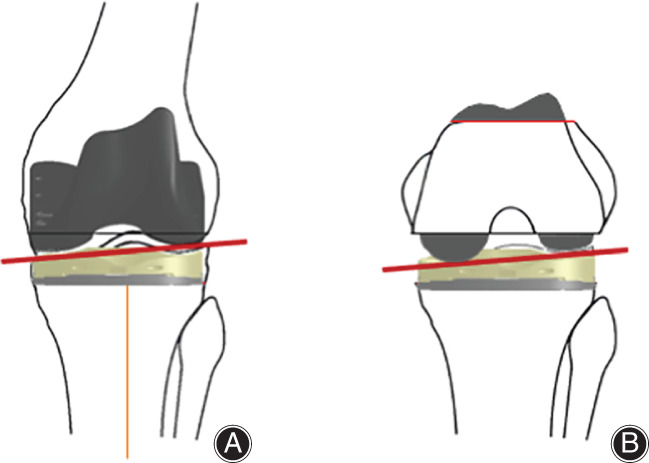
Schema of total knee arthroplasty with an anatomically shaped implant. (A) In extension, the proximal tibia and distal femur are resected at the same thickness as the lateral implants, respectively. The joint line is leveled to the original joint line with a medial inclination. (B) In flexion, the medial and lateral posterior condyles are resected at the same thickness as the medial and lateral condyles of the implant, respectively. As a result, the posterior joint line after implantation is leveled to the original posterior joint line.

In the second option, align the joint line of the tibia to the lateral joint surface and downsize the femur by 2 mm (Fig. 4, red line). In detail, the resection level of the proximal tibia is set to the same thickness as the lateral joint surface implant. The distal femoral resection is cut as thick as the implant from the lateral cartilage surface. This sets the joint line at the height of the lateral joint surface. Because it is unusual for the lateral posterior condyle resection to be as thick as the implant with the conventional posterior reference guide, downsizing by setting the bone cutting level anterior by 2 mm from the posterior reference guide is reasonable to adjust the posterior joint line to the lateral condyle level. This same situation frequently occurs in CR‐TKA, as with downsizing or using an implant with thin posterior condyles. In fact, some CR‐TKA systems use a 1 mm to 2 mm thinner posterior condyle compared to the bone resection to avoid flexion gap tightness due to this joint line inconsistency (i.e. NexGen CR‐flex and some other implants, as discussed later in Table 1). However, downsizing of the posterior condyle leads to decreased posterior condylar offset, causing a risk of decreased flexion range of motion^6^.

**TABLE 1:** Difference between resection thickness and implant thickness of the posterior condyle

	Resection thickness of posterior condyle in the posterior reference method, mm			Implant thickness of the posterior condyle, mm
	ER: 0°	ER: 3°, medial condyle	ER: 3°, lateral condyle	
Persona® CR	9	10.5	7.5	9
Attune® CR	9	10.5	7.5	8
Scorpio® CR	8	9	7	7.5
Triathlon® CR	8	9.5	6.5	8
Initia® CR	11	12.5	9.5	9
Journey® II CR	9.5	9.5	7	Medial: 9.5 Lateral: 7

ER: external rotation; Persona® (Zimmer Biomet Inc.); Attune® (Depuy Synthes, Warsaw, IN); Scorpio® (Stryker, Kalamazoo, MI); Triathlon® (Stryker); Initia® (Kyocera, Kyoto, Japan); Journey® (Smith & Nephew).

### When Using an Anatomically Shaped Implant (Fig. 4)

**Fig. 5:**
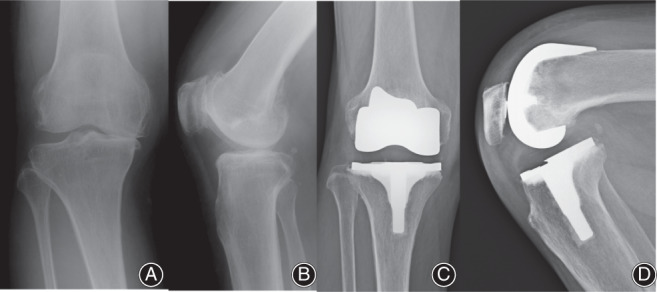
Radiographs of a right knee replaced with an anatomically shaped implant. (A) Preoperative anterior‐posterior view. (B) Preoperative lateral view. (C) Postoperative anterior‐posterior view. The joint line is maintained. (D) Postoperative lateral view.

The previously mentioned theory and procedure are for use with a conventional implant with the same thickness as the medial and lateral condyles in the mechanical alignment method. Most implants accept this condition, but some implants have an anatomic shape that mimics the physiologic articular surface, such as the Journey II (Smith & Nephew, plc., Watford, UK) and Fine (Teijin Nakashima Medical Co, Ltd., Okayama, Japan) implants. When these implants are used, the bone should be resected at the same thickness as the lateral part of the implant from the lateral joint surface in the proximal tibia and distal femur. This adjusts the medial joint line to be in physiologic position with a 3° medial inclination. When femoral sizing is performed with the posterior reference, the posterior condyle line matches the original joint line (Table 1). Although a limitation is that all knees are not at 3° varus and there are individual differences, the new joint line is less deviated from the original joint line than with a conventional implant (Fig. 5). Thus, the flexion gap is easier to match.

### Clinical Case Series Using an Anatomically Shaped Implant

In 30 consecutive patients with varus knee deformity (five male/15 female, mean age: 71.5 years), thickness of bone resection and intraoperative gap length were measured during TKA using Journey II CR. The proximal tibia was resected perpendicular to the mechanical axis for a thickness of 12 mm from the lateral joint surface (same thickness as the tibial component with the thinnest polyethylene insert of Journey II CR). After assessment of the joint gap, the distal femur was resected perpendicular to the femoral mechanical axis for a thickness of 8 mm from the lateral femoral surface. After removal of all the osteophytes, femoral bone resection was completed using the posterior reference method aligned in 3° external rotation to the posterior condylar axis. Ligament balancing was limited to the removal of osteophytes and release of deep layer of medial collateral ligament (MCL). The superficial layers of MCL and PCL were preserved. Thickness of resected bone was measured by a caliper, considering the thickness of the bone saw (1.5 mm) for medial and lateral side of distal femur, posterior condyle, and proximal tibia. Intraoperative joint gap was measured after the bone preparation in extension and flexion using a tensor device that independently measured the bone distance at medial and lateral side under a constant distraction force of 120 N. Table 2 shows the mean and standard deviation (SD) of the measurements. It shows that planned resection was performed, and the gap length was well‐balanced between extension and flexion. The maximum difference of the gap length between the medial extension gap and medial flexion gap was 3.5 mm, which was larger in extension gap. No patients had a flexion gap more than 1 mm smaller than the extension gap. The study protocol was reviewed and approved by the institutional ethical review board (approval number 4952). Informed consent was obtained using the opt‐out form on our center's website.

**TABLE 2:** Thickness of bone resection, polyethylene insert, and gap length during surgery using Journey II CR [*n* = 30, Mean (SD)]

	Thickness of bone resection, mm			Gap length, mm		Thickness of polyethylene insert, mm
	Distal femur	Posterior femur	Proximal tibia	Extension	Flexion	
Medial	8.3 (1.8)	10.1 (1.2)	6.2 (1.8)	21.5 (1.1)	21.6 (1.3)	10.0 (2.0)
Lateral	8.6 (1.8)	7.5 (1.7)	12.4 (1.3)	22.6 (1.5)	22.6 (1.8)	12.5 (2.0)

## Discussion

The standard surgical procedure for CR‐TKA includes measured resection techniques, which principally resect bone at the same thickness as the implant. Using this technique and the posterior reference method with conventional implants, the joint line heights between the proximal tibia and posterior femur do not match. This joint line discrepancy risks a tight flexion gap. It is important to individually consider how the joint line is altered at the medial and lateral sides of the proximal tibia, distal femur, and posterior femoral condyle.

Most surgical TKA instruments include a depth caliper to measure the resection level for the proximal tibia. The resection depth often is chosen at 10 mm, or 2 mm from the native cartilage or at the deepest point on the worn side, respectively. The 10‐mm resection from the lateral joint surface for a 10‐mm implant means that the postoperative joint line is set to the level of the lateral side. The distal femoral cut is usually set at the same thickness of the implant from the most distal end of the femur, which is usually on the worn medial side or the lateral cartilage. In most standard techniques, this means that the postoperative joint line is adjusted to the level of the lateral side in the coronal plane. In contrast, it is rare to primarily adjust the posterior joint line to the level of the lateral side with most TKA systems. This is why the flexion gap frequently becomes tight in CR‐TKA.

The most reasonable way to avoid this joint line discrepancy between the proximal tibia and posterior femoral condyle is to adjust the joint line to the center level of the medial and lateral joint lines (option 1). However, standard surgical instruments do not always allow the depth caliper to measure 1 mm to 2 mm deeper than the implant thickness for the tibial side. In such cases, surgeons can remove remaining cartilage from the lateral tibial surface to deepen the joint line for 1 mm to 2 mm. Standard instruments also do not have a way to resect the distal femur 1 mm to 2 mm thinner than the implant from the lateral side. Surgeons must manually estimate the bone thickness to be removed. The only method that most standard surgical instruments possess is downsizing the femur and placing it anteriorly on the posterior reference guide to reduce the posterior joint line to the same level as the lateral femoral condyle (option 2). This is usually used as an option when the sizing guide shows an in‐between size or when the tight flexion gap is encountered. However, some CR‐TKA systems adopt a 1 mm to 2 mm thinner posterior condyle compared to the thickness of bone resection to automatically downsize the posterior condyle (Table 1). The surgeon may be confused about whether they need to manually downsize the femoral component depending on the systems they use. Surgeons need to know the details of that information in advance.

This joint line theory method has some limitations. Posterior tibial inclination remains an important factor affecting the flexion gap and knee kinematics in CR‐TKA^7^, ^8^. Optimal posterior tibial inclination remains controversial and may vary depending on the TKA design and the patient. Preoperative deformities, including flexion contracture and severe constitutional varus, are factors affecting the extension gap. Therefore, the distal femoral joint line should sometimes be altered when the extension gap is insufficient in such cases. Nevertheless, this does not affect the flexion gap. Releases of MCL affects the extension gap and posterior gap. Excessive releases to the superficial layer of MCL should be avoided. However, with careful bone preparation considering the joint line theory, necessity of ligament release decreases.

## Conclusions

When the proximal tibia is removed from the lateral joint surface with the same thickness as the implant, the joint line on the proximal tibia is leveled at the height of the lateral side. This joint line setting may cause a tight flexion gap unless the posterior femoral joint line is set to the lateral condyle level. To prevent flexion gap tightness due to joint line discrepancy between the proximal tibia and posterior femoral condyle, the proximal tibia should be resected 1 mm to 2 mm thicker than the implant from the lateral surface. When using an implant with physiologic joint line inclination, the bone can be resected at the same thickness as the implant from the intact cartilage.

### Authorship Declaration

The author meets the authorship criteria according to the latest guidelines of the International Committee of Medical Journal Editors and that all authors are in agreement with the manuscript.

## Ethics Approval and Consent to Participate

The study protocol was reviewed and approved by the institutional ethical review board (approval number: 4952). Informed consent was obtained using the opt‐out form on our center's website.

## Availability of data and materials

The datasets used and/or analyzed during the current study are available from the corresponding author on reasonable request

## Funding

No financial support was obtained for this study.

## Author contributions

KO conceived the concepts, wrote the manuscript, performed surgery, and analyzed the data.

## Conflict of Interest

The author declares that they have no competing interests.
